# Free-Floating Mesothelial Cells in Pleural Fluid After Lung Surgery

**DOI:** 10.3389/fmed.2018.00089

**Published:** 2018-04-05

**Authors:** Arne Kienzle, Andrew B. Servais, Alexandra B. Ysasi, Barry C. Gibney, Cristian D. Valenzuela, Willi L. Wagner, Maximilian Ackermann, Steven J. Mentzer

**Affiliations:** ^1^Laboratory of Adaptive and Regenerative Biology, Brigham & Women’s Hospital, Harvard Medical School, Boston, MA, United States; ^2^Department of Diagnostic and Interventional Radiology, Translational Lung Research Center Heidelberg (TLRC), Member of German Center for Lung Research (DZL), University of Heidelberg, Heidelberg, Germany; ^3^Institute of Functional and Clinical Anatomy, University Medical Center of the Johannes Gutenberg-University Mainz, Mainz, Germany

**Keywords:** pleural fluid, mesothelial cells, pneumonectomy, lung regeneration, lung healing

## Abstract

**Objectives:**

The mesothelium, the surface layer of the heart, lung, bowel, liver, and tunica vaginalis, is a complex tissue implicated in organ-specific diseases and regenerative biology; however, the mechanism of mesothelial repair after surgical injury is unknown. Previous observations indicated seeding of denuded mesothelium by free-floating mesothelial cells may contribute to mesothelial healing. In this study, we investigated the prevalence of mesothelial cells in pleural fluid during the 7 days following pulmonary surgery.

**Study design:**

Flow cytometry was employed to study pleural fluid of 45 patients after lung resection or transplantation. We used histologically validated mesothelial markers (CD71 and WT1) to estimate the prevalence of mesothelial cells.

**Results:**

The viability of pleural fluid cells approached 100%. Leukocytes and mesothelial cells were identified in the pleural fluid within the first week after surgery. The leukocyte concentration was relatively stable at all time points. In contrast, mesothelial cells, identified by CD71 and WT1 peaked on POD3. The broad expression of CD71 molecule in postoperative pleural fluid suggests that many of the free-floating non-leukocyte cells were activated or proliferative mesothelial cells.

**Conclusion:**

We demonstrated that pleural fluid post lung surgery is a source of mesothelial cells; most of these cells appear to be viable and, as shown by CD71 staining, activated mesothelial cells. The observed peak of mesothelial cells on POD3 is consistent with a potential reparative role of free-floating mesothelial cells after pulmonary surgery.

## Introduction

The mesothelium, the surface layer of the heart, lung, bowel, liver, and tunica vaginalis, is a complex tissue implicated in both organ-specific diseases and regenerative biology (1, 2). An open question is the mechanism of mesothelial repair after injury (3). In a classic observation, Hertzler noted that the rate of mesothelial healing was independent of the size of the surface defect (4). This observation suggested that the typical mode of epithelial healing—namely, the centripetal migration of proliferating cells—was insufficient to account for mesothelial healing. These observations indicated that an alternative mechanism of mesothelial healing, such as the seeding of denuded mesothelium by free-floating mesothelial cells, may contribute to mesothelial healing (5–9).

Previous attempts to demonstrate free-floating mesothelial cells have used multiple animal models. Studies in rats have suggested that gentle rubbing of the liver surface results in the shedding of mesothelial cells (5). Also in rats, increased numbers of free-floating mesothelial cells have been found after trauma (8, 10, 11). In rabbits, spontaneous seeding of mesothelial cells on a fibrin-coated polyethylene sheet (12, 13) or diffusion chamber (6) have been observed. Despite these suggestive observations, the possibility of free-floating mesothelial cells remains controversial; specifically, the results in animal models have been inconsistent (14) and the few studies in humans have been limited to absolute cell numbers and malignant cells (15–17). Furthermore, both animal and human studies have been hampered by the lack of mesothelial cell-specific antibody probes capable of positively identifying mesothelial cells, while avoiding contamination with other mononuclear cells (18, 19).

Our hypothesis was that if free-floating mesothelial cells contribute to mesothelial healing, then we should find mesothelial cells in pleural fluid after pulmonary surgery. To test this hypothesis, we studied the pleural fluid of 45 patients after lung transplantation or lung resection. Free-floating mesothelial cells were identified by anti-CD71 and anti-WT1 antibodies and flow cytometry. Anti-WT1 antibodies recognize the WT1 (Wilms’ tumor 1) gene product which appears to be crucial for the development of several organs and tissues including the mesothelium (20, 21). Because of the inconsistent nuclear and perinuclear distribution of the WT1 protein, however, the staining of the anti-WT1 can be variable (22). A useful complement to anti-WT1 was the anti-CD71 antibody. CD71, also known as the transferrin receptor, has been recently shown to discriminate mesothelial cells from contaminating CD45^−^ mononuclear cells in flow cytometry (23). Further, the expression of CD71 appears to increase with cellular activation (24).

In this report, we used anti-WT1 and anti-CD71 staining and flow cytometry to identify mesothelial cells in the pleural fluid after pulmonary surgery. The peak of mesothelial cells 3 days after surgery is consistent with a potential reparative role for free-floating mesothelial cells after pulmonary surgery.

## Materials and Methods

### Patients

Institutional Review Board approval was obtained from Brigham & Women’s Hospital. Informed consent was obtained from all patients for the anonymized use of discarded tissue and fluid. Pleural effusion fluid was collected from patients after partial lung resection or transplantation at different post-operative time points. Fluid was obtained from standard Pleur-Evac drain systems (Teleflex, Morrisville, NC, USA). We studied a total of 45 patients with pleural drains. The average sample volume was 18.9 ml and the minimum sample volume was 4 ml. Human pleura were obtained from the Brigham & Women’s Hospital Tissue and Blood Repository. No patient identifiers or medical information were recorded.

### Cell Preparation

Pleural effusion fluid samples were diluted using PBS. The mononuclear cell layer obtained from Ficoll-Paque isolation was washed two times in ice-cold FACS buffer (PBS with 0.5% BSA, 0.1% NaN_3_ sodium azide). For optimal antibody concentrations cells were counted in a hemocytometer using trypan blue and diluted to 1–5 million cells per ml. After staining, cells were stored in 4% PFA in FACS buffer at 4°C in the dark until flow cytometry analysis.

### Cell Viability

In all patients, cell viability was determined by trypan blue exclusion. When appropriate, cell viability of pleural fluid samples was confirmed using calcein-AM and ethidium homodimer-1 (LIVE/DEAD™ Viability Kit, Thermo Fischer, L3224). Samples were incubated with 2 uM calcein-AM and 4 uM ethidium homodimer-1 working solution for 30 min at 27°C. Green-fluorescent calcein-AM staining indicated intracellular esterase activity in live cells, while red-fluorescent ethidium homodimer-1 indicated the loss of plasma membrane integrity in dead cells. After washing the samples twice with PBS, pleural fluid sample smears were analyzed using stage scan fluorescent microscopy, and cell numbers were quantified.

### Monoclonal Antibodies

All antibodies were obtained from commercial sources. Staining for CD45 [FITC conjugated mouse monoclonal (B-A11), Abcam, ab27287] and CD71 [AlexaFluor647 conjugated mouse monoclonal (MEM75), Abcam, ab187777] was performed on ice for 30 min each. Additional WT-1 (1/50 dilution; rabbit anti-human, Abcam, ab15249) staining was performed on ice for 30 min after permeabilization with 0.1% Tween-PBS and blocking with 10% goat serum in FACS buffer. For flow cytometry, AlexaFluor405 goat anti-rabbit antibody (1/2,000 dilution; Abcam, ab175655) was used as a secondary antibody. For fluorescence histochemistry, FITC goat anti-rabbit antibody (1/100 dilution; Thermo Fischer, F-2765) was used as a secondary antibody to allow for Hoechst staining of the sections.

### Flow Cytometry

Flow cytometry analysis was performed on LSRFortessa (BD Bioscience, Mountain View, CA, USA). All data were analyzed using FCS Express 5 software (*De Novo* Software, Los Angeles, CA, USA). Gating was performed by comparing the fluorescence intensity of stained cell markers and physical cell parameters using side- and forward-scatter of stained samples and isotype controls.

### Fluorescence Histochemistry

Human lung specimens were obtained from the Brigham & Women’s Hospital Tissue and Blood Repository after processing according to hospital IRB procedures. The anonymized samples were fixed in 4% paraformaldehyde in PBS at 4°C for 24 h. After 24 h, the specimens were submerged in O.C.T. compound and frozen in a mixture of acetone and dry ice. The O.C.T. blocks were kept at −80°C for 24 h prior to cryosectioning. Cryostat sections were obtained from human lung specimens embedded in O.C.T. compound, and snap frozen. After warming the slide to 27°C, the sections were fixed and permeabilized in acetone at 4°C. The slides were washed with PBS buffer and blocked with 10% goat serum in PBS for 30 min. The slides were treated with primary and secondary antibody. The slides were incubated with each antibody for 1 hour at 27°C, washed three times, counterstained with Hoechst 33342 (Sigma-Aldrich, St. Louis, MO, USA) for 15 min and mounted using VectaShield mounting media (Vector Laboratories, Inc., Burlingame, CA, USA).

### Statistics

The unpaired Student’s *t*-test for samples of unequal variances was used to calculate statistical significance. The data was expressed as mean ± 1 SD. The significance level for the sample distribution was defined as *p* < 0.05.

## Results

### Pleural Fluid Dynamics

**Figure 1:**
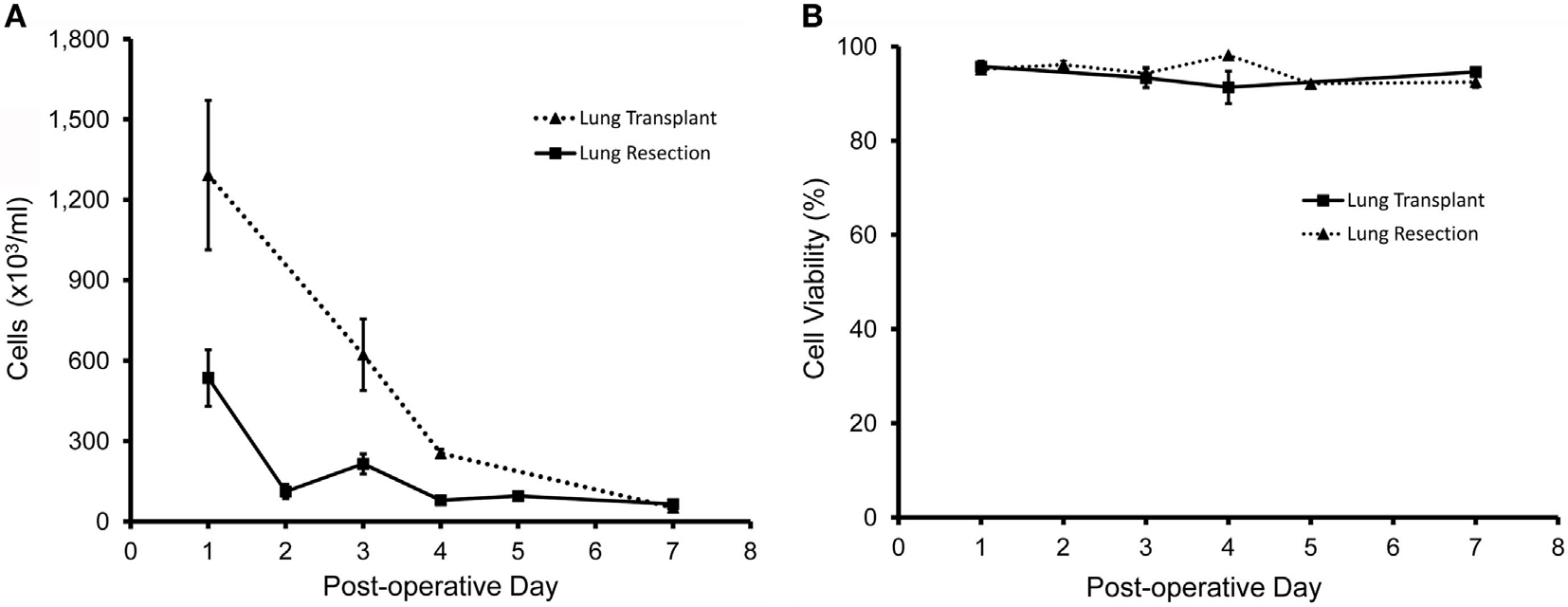
Cellular content of pleural fluid after lung resection (solid line) and lung transplantation (dotted line). When clinically available, samples were obtained from *N* = 45 patients; *N* = 37 patients after lung resection; and *N* = 8 patients after lung transplantation. **(A)** After red cell lysis, cell concentrations were determined by manual counting with a hematocytometer. **(B)** Cell viability was determined by light microscopy and trypan blue exclusion (27) as well as stage scan fluorescent microscopy after calcein-AM and ethidium homodimer-1 cell viability staining. Error bars reflect ± 1 SD.

**Figure 2:**
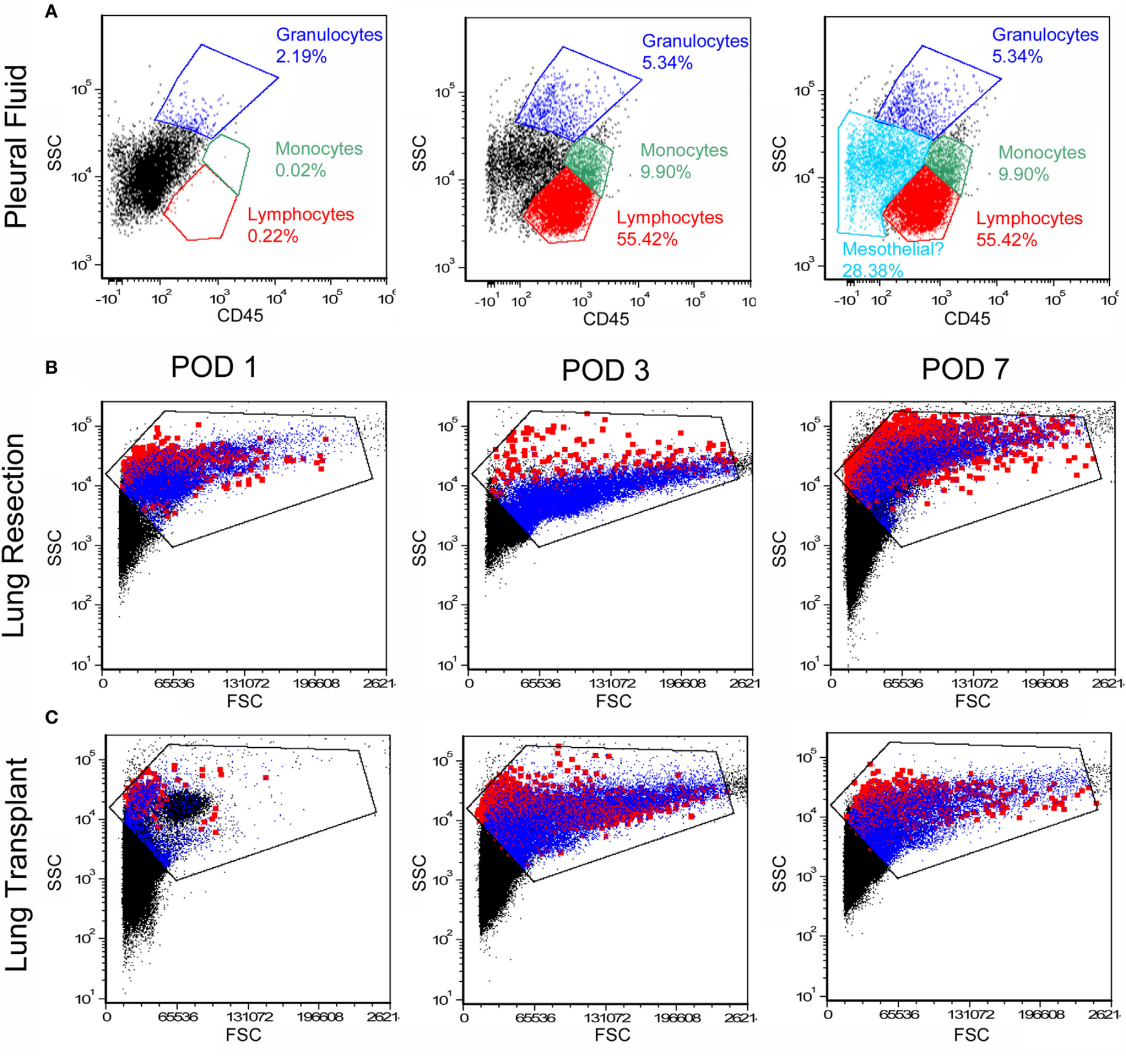
Flow cytometry profile of cells in the pleural fluid. After exclusion of debris and red cells, the remaining cells were studied by flow cytometry. **(A)** Anti-CD45 staining and side-scatter (SSC) analysis demonstrated a dominant mononuclear population. A representative sample on day 1 after surgery demonstrated 70% mononuclear cells and 5% granulocytes; the remaining 25% of cells were compatible with mesothelial cells. Unstained negative controls were compared to samples stained with detection antibodies **(B,C)**. More detailed size analysis with forward light scatter (FSC) on postoperative day 1, 3, and 7 showed no consistent size relationship between CD45^+^ cells (blue) and CD45^−^ cells (red; the *Emphasize Plot Tool*, FCS Express, was used for data presentation). Representative histograms are shown.

Pleural fluid was sampled after both lung transplantation and pulmonary resection using standard collection chambers. All pleural fluid samples had a volume of ≥4 ml and the average sample volume was 18.9 ml. As expected, the number of pleural fluid cells was maximal on postoperative day 1 and gradually declined over the first week (Figure 1A). Viability of the cells approached 100% (Figure 1B). Flow cytometry of the pleural fluid cells after labeling with the leukocyte marker anti-CD45 indicated that 75% of the cells were leukocytes comprising granulocytes, monocytes, and lymphocytes; approximately 25% of the pleural fluid cells on postoperative day 1 were non-leukocytes (Figure 2A). Size analysis based on flow cytometry forward light scatter (25, 26) demonstrated that the leukocyte and non-leukocyte cell populations were distributed throughout the size spectrum (Figures 2B,C).

### Human Mesothelial Marker

**Figure 3:**
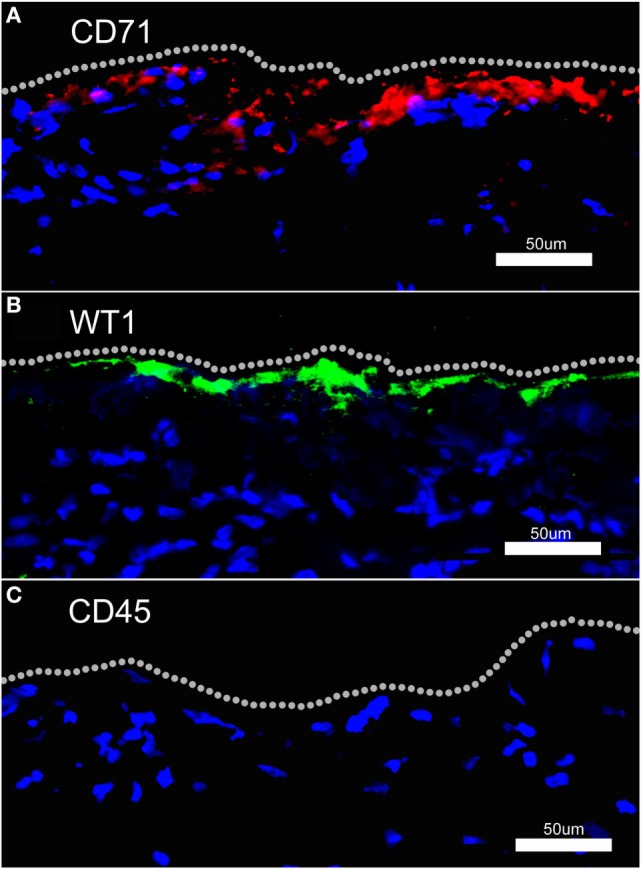
Fluorescence immunohistochemistry of human pleural mesothelium. Human pleura, obtained from surgical specimens, was stained with fluorochrome-labeled anti-CD71 (A), anti-WT1 (B), and anti-CD45 (C) monoclonal antibodies and counterstained with Hoechst 33342. Both anti-CD71 and anti-WT1 antibodies demonstrated discontinuous staining of the pleural mesothelium. There was no detectable pleural staining with labeled anti-CD45 monoclonal antibody. Bar = 50 um.

**Figure 4:**
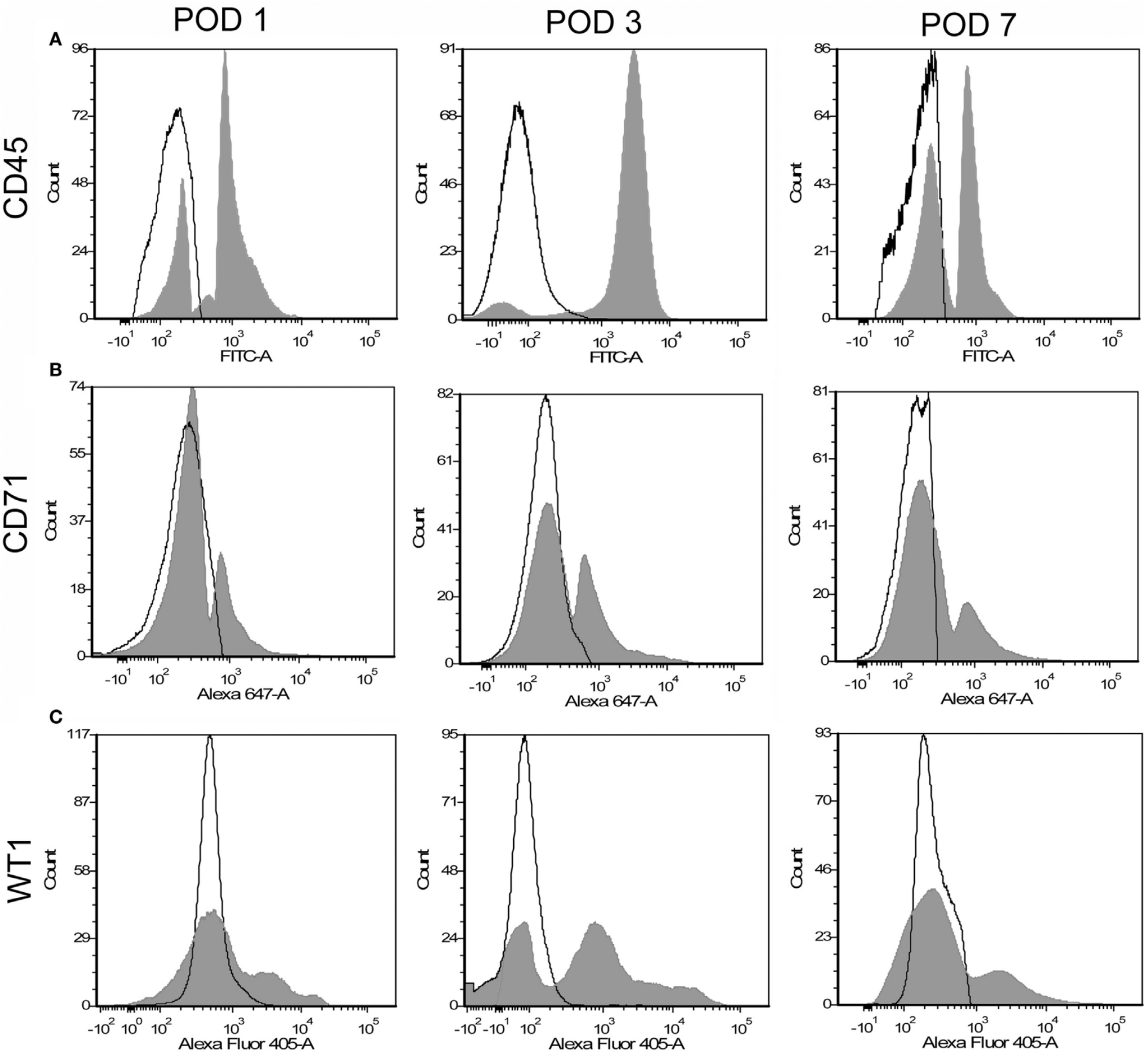
Flow cytometry profile of pleural fluid cells after surgical resection. Representative single parameter histograms of CD45^+^ **(A)**, CD71^+^ **(B)**, and WT1^+^ **(C)** cells at three time points after surgery: postoperative days (POD) 1, 3, and 7 (gray). Negative controls (white) reflect samples without detection antibody.

To identify potential mesothelial markers, we immunostained human mesothelium with anti-CD71 and anti-WT1 monoclonal antibodies. Both antibodies demonstrated prominent, albeit discontinuous, staining of the pleural mesothelium with little background lung parenchymal staining (Figures 3A,B). In contrast, anti-CD45 monoclonal antibody did not stain the pleura (Figure 3C). Using these antibodies, flow cytometry over the first week after lung surgery demonstrated that leukocytes were the predominant cell type (Figure 4A). Both the CD71 (Figure 4B) and the WT1 (Figure 4C) cell populations peaked on postoperative day 3.

### Mesothelial Cell Dynamics

**Figure 5:**
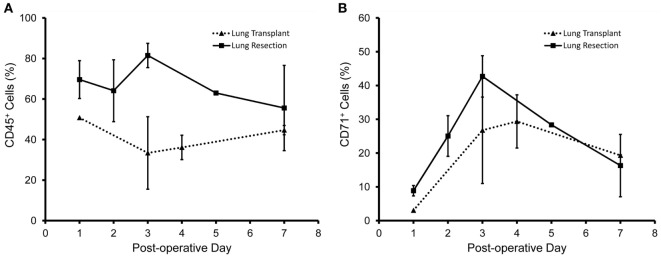
Time course of pleural fluid cell populations after surgery. Pleural fluid obtained from patients after lung resection (solid line) and lung transplant (dotted line) are shown. **(A)** Leukocytes, identified by anti-CD45 antibodies, were a relatively consistent percentage of cells during the first week after surgery. **(B)** In contrast, the percentage of CD71^+^ cells peaked 3 days after surgery. Data based on *N* = 40 patients. Error bars reflect ± 1 SD.

**Figure 6:**
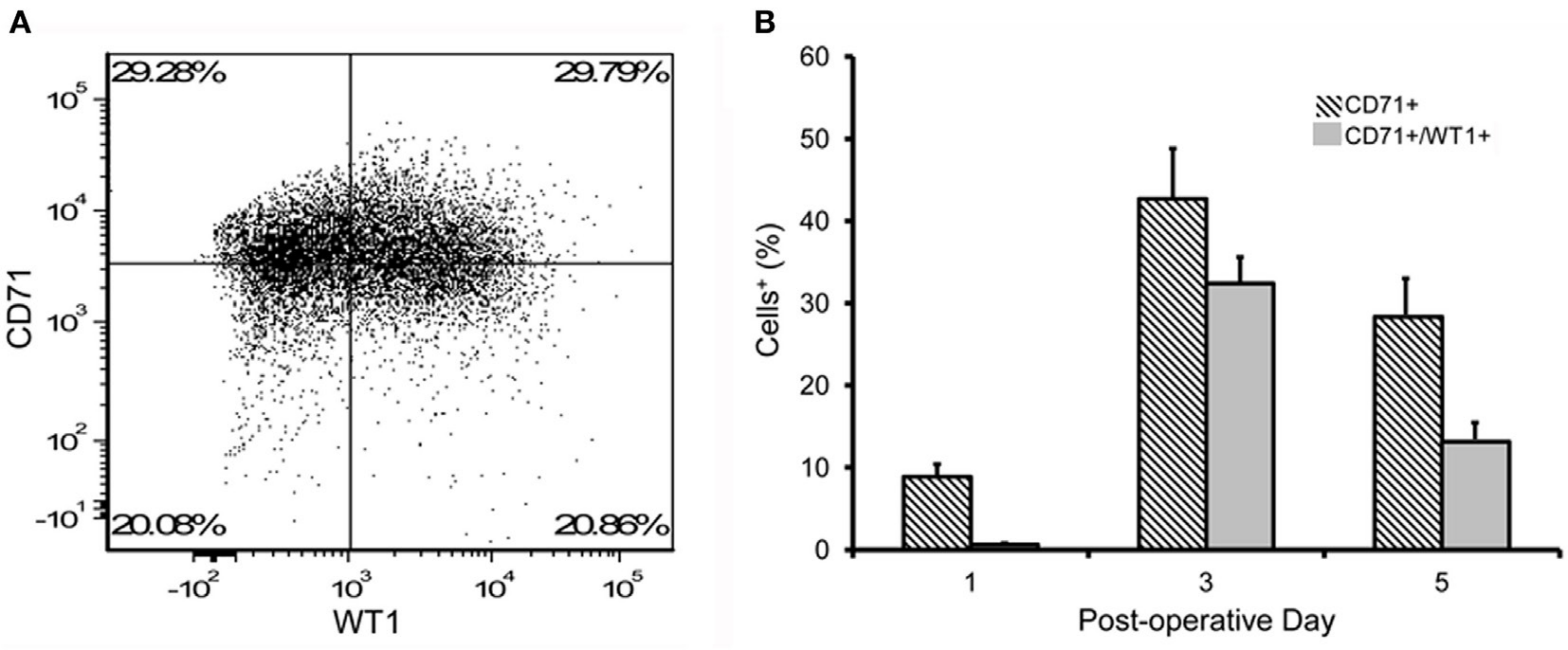
Flow cytometry of pleural fluid CD45^−^ cells. **(A)** Representative dual parameter histograms of CD45^−^ cells on postoperative day 3 demonstrated 70% of cells were positive for CD71 and/or WT1; 35% of cells were positive for both CD71 and WT1. **(B)** Consistent with single parameter staining, the double parameter flow cytometry profiles demonstrated a peak concentration of presumed mesothelial cells on postoperative day 3. *N* = 21 patients. Error bars reflect ± 1 SD.

The concentration of CD45^+^ cells was relatively stable over the first week (Figure 5A). In contrast, the CD71^+^ population peaked on postoperative day 3 in both the lung resection and lung transplant patients (Figure 5B). Using dual parameter flow cytometry and both mesothelial markers, 70% of the CD45^−^ pleural cells stained with either CD71 or WT1 monoclonal antibodies; 35% of cells were positive for both CD71 and WT1 (Figure 6A). The concentration of both single and double positive cells peaked on postoperative day 3 (Figure 6B).

## Discussion

In this report, we studied the pleural fluid of 45 patients after lung transplant and pulmonary resection. The pleural fluid cells demonstrated several characteristics: (1) near-100% cell viability, (2) a dominant population of CD45^+^ leukocytes (75%) peaking on postoperative day 1, and (3) a smaller population of CD45^−^ cells (25%) peaking on postoperative day 3. Flow cytometry using the mesothelial markers CD71 and WT1 demonstrated a phenotype consistent with both activated and unactivated mesothelial cells. Further, forward light scatter indicated that the presumed mesothelial cells reflected a broad size spectrum. We conclude that free-floating pleural cells, reflecting both activated and unactivated mesothelial cells, are present in the pleural fluid after lung surgery.

Because of the important clinical implications of malignant pleural effusions (28), most human studies of pleural fluid cells have focused on the identification of markers for malignant cells (17, 23, 29). The potential utility of CD71 as a marker of benign mesothelial cells was a byproduct of these studies (23). The CD71 cell surface molecule, otherwise known as the transferrin receptor, is expressed on most proliferating normal and transformed cells (30). CD71 also binds and internalizes the iron-loaded ligand transferrin (31). The broad expression of CD71 in the postoperative pleura suggests that many of the free-floating non-leukocyte cells were activated or proliferative mesothelium. The identity of these cells as activated mesothelial cells was supported by staining with the established mesothelial marker WT1 (22, 32).

A challenge for *in vivo* studies of mesothelium is the absence of a reliable canonical marker of mesothelial cells. Although additional markers, such as mesothelin (33), GPM6a (34), and CD200 (35) have been proposed, these markers label a subset of the mesothelial cell population. The variable staining reflects either cell at different stages of activation or different subpopulations of mesothelial cells (36). The possibility of distinct populations of pleural mesothelial cells is underscored by the recent descriptions of pleural mesothelial–mesenchymal transition after murine pneumonectomy (37). In response to this variability, we designed our flow cytometry experiments using both anti-CD71 and anti-WT1 antibodies to optimize our detection of the potential mesothelial cell population.

A limitation of human studies is the difficulty in estimating the absolute number of free-floating mesothelial cells available for seeding injured mesothelium. Despite variability in cell numbers, cell viability was nearly 100% indicating that the cells were not dying exfoliated cells, but mesothelial cells were capable of participating in mesothelial repair. The expression of the activation marker CD71 suggests that many of these cells were metabolically activated (38, 39). Based on cell surface area calculations derived from scanning electron microscopy morphometry of nonreactive human mesothelium, we estimate that the post-operative day 3 pleural fluid contains sufficient numbers of activated mesothelial cells to cover several cm^2^ of denuded mesothelium (40). Furthermore, we speculate that the increased pleural fluid noted after lung surgery functions not only as a vehicle for cell distribution, but also as a nutrient source for free-floating cells (41).

An interesting observation was the peak concentration of free-floating mesothelial cells 3 days after surgery. Postoperative day 3 is within the 7-day timeframe for pleural healing noted by many surgical studies (42–44). In addition, 3 days is the peak of the epithelial–mesenchymal transition noted after murine pneumonectomy (37). In Ysasi et al. scanning electron microscopy demonstrated pleural transitional cells without the cell–cell and cell-substratum adhesions characteristic of mesothelium (37). Whereas some of these cells demonstrably migrated into the lung parenchyma (37), it is equally plausible that other cells were released into the pleural fluid. Elegant labeling studies in rats have demonstrated that free-floating mesothelial cells, in preference to cultured fibroblasts, bind to wounded mesothelium (9).

The observations in this study also have implications for future investigation. We have demonstrated that the pleural fluid post lung surgery is a source of mesothelial cells; most of these cells appear to be activated mesothelial cells. The high viability of these cells and the convenient drainage chambers used after lung surgery suggests an opportunity for *in vitro* studies. We anticipate that these cells will provide an opportunity to define more comprehensive markers of human mesothelium as well as an opportunity to explore the proliferative and secretory activity of human mesothelium. In lung transplant patients, any mismatch between HLA antigens would provide an opportunity to distinguish between a visceral (donor) or parietal (host) pleural origin of the free-floating cells. The influence of size matching (parenchymal stretch) and ischemia could also be explored after lung transplantation.

## Ethics Statement

Institutional Review Board approval was obtained from Brigham & Women’s Hospital. Informed consent was obtained from all patients for the anonymized use of discarded tissue and fluid. Pleural effusion fluid was collected from patients after partial lung resection or transplantation at different post-operative time points. Fluid was obtained from standard Pleur-Evac drain systems (Teleflex, Morrisville, NC, USA). We studied a total of 45 patients with pleural drains. The average sample volume was 18.9 ml and the minimum sample volume was 4 ml. Human pleura were obtained from the Brigham & Women’s Hospital Tissue and Blood Repository. No patient identifiers or medical information were recorded.

## Author Contributions

AK, AS, AY, BG, CV, WW, MA, and SM designed the study and developed the methodology. AK and BG acquired the patient samples. AK performed the experiments. AK, AY, and SM analyzed the data. MA and SM supervised the findings of this work. All authors discussed the results and contributed to the final manuscript.

## Conflict of Interest Statement

The authors declare that the research was conducted in the absence of any commercial or financial relationships that could be construed as a potential conflict of interest. The reviewer DC and the handling Editor declared their shared affiliation.

## Abbreviations

POD, postoperative day; PBS, phosphate-buffered saline; FACS, fluorescence-activated cell sorting; CD, cluster of differentiation; PFA, paraformaldehyde; calcein-AM, calcein-acetoxymethyl; OCT compound, optimal cutting temperature compound; HLA, human leukocyte antigen; WT1, Wilms tumor protein.
